# Nasal Emulgel’s Role in Preventing Coronavirus Infection

**DOI:** 10.3390/pharmaceutics17060795

**Published:** 2025-06-19

**Authors:** Francesca Accioni, Giovanna Rassu, Antonio Brunetti, Erika Plicanti, Giulia Freer, Antonio Carta, Paolo Giunchedi, Elisabetta Gavini

**Affiliations:** 1 Department of Medicine, Surgery and Pharmacy, University of Sassari, 07100 Sassari, Italy; francesca.accioni@gmail.com (F.A.); acarta@uniss.it (A.C.); pgiunc@uniss.it (P.G.); eligav@uniss.it (E.G.); 2 Biomedical Sciences Department, University of Sassari, 07100 Sassari, Italy; brunetti@uniss.it; 3 Department of Medical Biotechnologies, University of Siena, 53100 Siena, Italy; e.plicanti@studenti.unipi.it; 4 Centro Retrovirus, Department of Translational Research, University of Pisa, 56127 Pisa, Italy; giulia.freer@unipi.it

**Keywords:** Mucoadhesive emulgel, Nasal formulation, Barrier to cell-to cell spread, anti-SARS-CoV-2, Pandemic prevention

## Abstract

**Background/Objectives:** Coronaviruses (CoVs) are a large family of respiratory viruses that cause respiratory illnesses ranging from mild colds to severe diseases such as severe acute respiratory syndrome and pandemics. The nasal cavity is a primary site for CoV entry and transmission. The study aimed to prepare a novel mucoadhesive emulgel specifically formulated with simple, safe, and cost-effective excipients to create a barrier on the nasal mucosa that impedes CoV infection. This formulation strategy was specifically designed to enable rapid and straightforward in vivo translation, addressing a critical gap in preventive measures against respiratory viruses. **Methods**: Three emulgels, containing macadamia oil, Carbopol and different percentages of hydroxypropyl methylcellulose (1, 1.2 and 1.5% (w/v), HPMC), were properly prepared and characterized for mucoadhesion, viscosity, and spreadability. The biological activity against SARS-CoV-2 was evaluated in vitro on infected epithelial cells. **Results**: The emulgel with 1.2% HPMC demonstrated optimal physicochemical properties (mucoadhesion: 342 ± 9 mN/cm^2^; viscosity: 1080 ± 83 cp; spreadability: 7.27 ± 0.06 cm) suitable for nasal application. Importantly, in vitro biological assays demonstrated that this emulgel significantly inhibits SARS-CoV-2 infection in epithelial cells, indicating an effective barrier to viral diffusion. **Conclusions**: By employing readily available, safe, and inexpensive excipients, this novel mucoadhesive emulgel offers a practical, scalable, and rapidly translatable nasal prophylactic approach to prevent early SARS-CoV-2 infection, addressing a critical unmet need in pandemic preparedness.

## 1. Introduction

Coronaviruses (CoVs) are a group of respiratory viruses recognized as leading causes of respiratory infections; they can cause mild to severe disease by infecting cells in the upper respiratory tract, bronchial epithelium and lungs, with the most recently discovered being notably more pathogenic and capable of causing severe or even life-threatening respiratory conditions [1]. Among these, since its first appearance in late 2019, severe acute respiratory syndrome coronavirus 2 (SARS-CoV-2) has remained a significant challenge to human health. Despite the increasing progress achieved both in the field of vaccines/new drugs effective against SARS-CoV-2 [2] or anti-influenza entry inhibitors [3], researchers have focused on finding strategies for preventing the viral infection in its early stages since only a few of the currently available drugs have been successful at treating SARS-CoV-2 [4,5,6] or did not induce drug resistance during influenza pandemics [3]. In fact, prevention has been key in avoiding the transmission of severe acute respiratory syndrome corona-virus 2 (SARS-CoV-2), which is considered the source of the coronavirus disease (COVID-19) pandemic.

The receptor angiotensin-converting enzyme-2 (ACE2) has been considered the most important mediator for the entrance of the SARS-CoV-2 virus into the host cell, and it is highly expressed on nasal epithelium [4]. ACE2 was described for the first time in 2003 as the critical target for the entry of SARS-CoV, the virus responsible for the SARS 2002–2004 epidemic. It was also studied as the receptor of alphacoronavirus HCoV-NL63 that causes mild upper respiratory tract infections [5]. Several animal models within human transcriptome databases demonstrated that even if ACE2 expression in the lower lung is primarily restricted to type II alveolar cells, it is more abundant in the upper bronchial epithelia and significantly more expressed in the nasal epithelium, particularly in the ciliated cells [6,7,8]. SARS-CoV-2 infection gradient reflects the variation in ACE2 expression level in the respiratory tract, with nasal ciliated cells serving as the main targets for SARS-CoV-2 replication in the early stages of infection [1,5]. The Huh-7 cells are a recognized model for SARS-CoV-2 infection and ACE-2 is expressed in this cell line, as proved and reported by Niet et al. [9] and by Sherman et al. [10]. The Vero TMPRSS cells are stably transfected with the TMPRSS protease, making them a highly permissive in vitro model for SARS-CoV-2.

Considering all these facts, a newly developed novel nasal formulation has been considered an interesting alternative as a selective approach that is able to block SARS-CoV-2 transmission in the first stages [11,12,13]. Nasal sprays for the treatment of respiratory infections such as common cold or influenza that already are commercially available have been evaluated as a potential approach to prevent SARS-CoV-2 [13,14,15,16]. The strategy of action has been based on the incorporation of small well-known species such as reactive oxygen and nitric oxide that actively affect the virus [17]. Recently, a study reported a computational approach to quantify the protection rate resulting from a wide range of delivery parameters to develop a targeted nasal spray as a barrier to impede the viral entry of common flu and SARS-CoV-2 viruses [18]. The main limiting factor of the nasal formulation is the reduced long-lasting activity correlated with mucociliary clearance and cilia beating that move the mucous blanket towards the nasopharynx with a speed in the range of 3–25 mm/min [19]. Hence, a formulation, if not properly tailored, will be removed from the nasal cavity in about 15 min [20].

**Figure 1:**
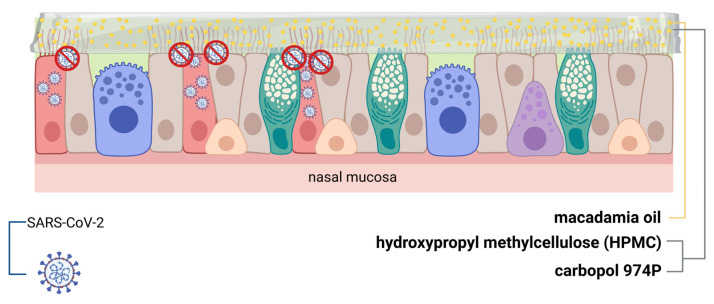
Mechanism of action of the emulgel nasal spray against SARS-CoV-2. Created in BioRender. Langellotto, M. (2025).

The aim of this work was to develop a novel nasal emulgel designed to form a functional barrier on the nasal mucosa, thereby impeding the initial replication of SARS-CoV-2 virus (Figure 1). Emulgels have been considered as an emerging nasal formulation due to their good rheological properties, which offer a prolonged residence time on the mucosal surface [21,22,23]. Researchers have reported the positive features of both gels and emulsions for the topical delivery of many different hydrophilic and lipophilic drugs, demonstrating long-lasting stability due to the addition of the gel to the emulsion [24,25,26]. The common oils employed in emulgel compositions are sesame oil, balsam oil, mineral oil, liquid paraffin, birch oil, isopropyl palmitate, castor oil, isopropyl myristate, myrrh oil, and rosehip oil [27]. Recently, Asif et al. reported a possible role of chemo-herbals and essential oils as effective approaches to fight COVID-19 for their antiviral, anti-inflammatory, and immunomodulatory properties [28]. Macadamia oil has been considered a natural antioxidant since it contains tocotrienols, tocopherol (vitamin E), and oleic acid considered natural antioxidants able to significantly reduce inflammation and oxidative stress [29,30]. Compounds that both interfere with this process, avoiding severe symptoms, and block the mechanism of entry have been confirmed as comprehensive tools to control the infection [31].

Polymers with gelling and mucoadhesive activity have been widely employed for the formation of semi-solid networks such as emulgels [27]. In addition, since at very low concentrations these agents allow the creation of a semi-solid texture that reduces the clearance rate and performs residence time at the target site [27], they were employed for the in loco/ex loco formation of a physical barrier to stop the entry mechanism [11,12,13,32]. Hydroxypropyl methylcellulose (HPMC) is a semi-synthetic, viscoelastic, mucoadhesive and inert polymer widely used in intranasal delivery as a rate-controlled polymer with barrier-enforcing properties [33,34,35]. Carbopol is the most widely employed polymer in the preparation of gels as a mucoadhesive agent due to its outstanding mucoadhesive behavior and useful viscosity [36].

In contrast to existing nasal formulations, this new emulgel uniquely combines macadamia oil, carbopol and optimized concentrations of HPMC. The excipients have been specifically chosen because they are simple, safe and inexpensive, ensuring quick and easy in vivo application of the emulgel. This addresses the lack of effective preventive measures against respiratory viruses. Three emulgels containing different HPMC concentrations were prepared and characterized in terms of physical appearance, morphology, pH, viscosity, spreadability, and mucoadhesive strength for evaluating the optimal properties for nasal administration. The efficacy of the lead emulgel formulation in preventing SARS-CoV-2 infection was rigorously assessed using two well-established and highly relevant in vitro models: Huh-7 cells and Vero-TMPRSS2 cells. These cell lines were specifically selected due to their widespread recognition in SARS-CoV-2 research and their confirmed expression of the ACE2 receptor, critical for viral infection [9,10]. Selecting these specific cell lines offers the closest approximation to ensure that the emulgel’s inhibitory effects on viral replication are predictive of its in vivo potential.

## 2. Materials and Methods

### 2.1. Materials

Methocel™ K4M Hydroxypropyl Methylcellulose (HPMC) was supplied from Dow Chemical Company Limited (Derbyshire, UK). Carbopol^®^ 974P NF polymer was provided by Lubrizol (Wickliffe, OH, USA). Macadamia oil was purchased from Farmalabor (Canosa di Puglia, Italy). Ultrapure water was obtained by the MilliQ R4 system from Millipore (Milan, Italy). Phosphate-buffered saline (PBS, NaCl 0.138 M; KCl 0.0027 M; pH 7.4, at 25 °C), Tween 80 and mucin from porcine stomach (Type II) were obtained from Sigma-Aldrich (Milan, Italy). Dulbecco’s Modified Eagle’s Medium (DMEM), fetal bovine serum (FBS) and L-glutamine were purchased from Life Technologies (Milan, Italy). Alamar blue was purchased from Merck (Milan, Italy).

### 2.2. Preparation of the Nasal Emulgels

Three different emulgels, with various percentages of HPMC, were prepared by following the method proposed by Khan et al. [37] but appropriately modified to disperse the components. Thus, the emulgels formed with 0.2% (w/v) Carbopol and 1% (w/v) of HPMC (BF 1) or 1.2% (w/v) of HPMC (BF 1.2) or 1.5% (w/v) of HPMC (BF 1.5) were obtained by dispersing the polymers in the water phase with constant stirring to form the homogenous network. Afterward, 2 mL of macadamia oil was added to 8 mL of the gel mixture, vortex-shaken (2 min), and homogenized using an UltraTurrax T-25 digital (IKA, Staufen, Germany) for 30 s at 16,000 rpm.

### 2.3. Characterization of the Nasal Emulgels

The physical appearance of the three formulations was observed to evaluate basic parameters such as color/color change, homogeneity, and creaming/phase separation [37]. Moreover, the morphology of BF 1.2 were observed by Leica DM6 polarized light microscopy (stack 10×) (Leica microsystems, Wetzlar, Germany) [38].

The pH of BF 1, BF 1.2 or BF 1.5 was measured by a pH meter B417 (Hanna Instruments, Padova, Italy).

The viscosity of BF 1, BF 1.2 or BF 1.5 was determined at 25 °C by following a modified method previously reported by Khan et al. [37]. The viscometer Fungilab™ Alpha Series R Model Rotational Viscometer (Fungilab Inc., Hauppauge, NY, USA) was used with spindle no. 3. The spindle was dropped vertically into the center of beaker filled with a proper amount of sample, avoiding touching the glass bottom. The spindle was rotated at 100 rpm for 2 min prior reading the results. The analysis was performed in triplicate for three different formulations (*n* = 9; ±standard deviation, SD).

Spreadability was measured by following a method previously reported [39,40], as shown in Figure S1. In particular, 1 g of BF 1, BF 1.2 or BF 1.5 was placed within the circle of 1 cm diameter pre-marked on a glass plate, covered by a second glass plate. A weight of 500 g was allowed to rest for 5 min. The increase in the diameter related to sample spreading was reported (*n* = 9; ±standard deviation, SD). The correlation between the obtained data of viscosity, spreadabilty and the three percentages of HPMC were investigated by statistical analysis (Section 2.6).

Mucoadhesive properties of the emulgels were estimated using a modified precision balance [41,42] by measuring the detachment forces between the emulgels and a paper filter conditioned with 2% mucin solution [43] 120 s after the contact was established. Mucoadhesive strengths were expressed as detachment force (in mN/cm^2^) employed to detach the two systems. The measurements were performed in triplicate for the three formulations.

### 2.4. Physical Stability

Stability of the three emulgels was examined at temperatures of 25 °C for 28 days [37]. The samples were kept in amber glass vials closed with a screw cap, and every 7 days evaluated for appearance, pH, and viscosity.

### 2.5. Biological Assays

#### 2.5.1. Cell Culture and Treatments

Huh-7 cells (naturally expressing ACE-2 and TMPRSS) cells were purchased from American Type Culture Collection (Manassas, VA, USA) and were cultured at 37 °C and 5% CO_2_ in DMEM supplemented with 7% of fetal bovine serum (FBS) and 2 mM L-glutamine. Vero-TMPRSS were cultured in a similar manner to Huh-7 cells, with 1 mg/mL of G418 (Merck) added once a month [44]. Different concentrations of BF 1.2 were used to treat Huh-7 and Vero-TMPRSS cells 1.5 h after viral infection. All cell cultures were tested for mycoplasma contamination [45]. All biological experiments were repeated in triplicate (*n* = 3; ±standard deviation, SD).

#### 2.5.2. Cell Viability Assay

Cell viability assays were performed as follows: Huh-7 and Vero-TMPRSS cells were plated (10^3^ cells/well) in 96-well plates and incubated overnight. Then, cells were incubated with serial dilutions (from 330 to 0.6 µM) of BF 1.2 for 48 h, and their viability was assessed using WST (Merck, Darmstadt, Germany) assay according to the manufacturer’s instruction. Cell viability was also assessed using the Operetta CLS high-content imaging device (PerkinElmer, Hamburg, Germany) by counting the nuclei of uninfected cells treated with different concentrations of BF 1.2 and comparing the nucleus count to that of untreated cells.

#### 2.5.3. SARS-CoV-2 Infection

The infection of Huh-7 and Vero-TMPRSS cells was performed with 0.1 MOI of SARS-CoV-2 isolate SARS-CoV-2/Human/ITA/PAVIA10734/2020, clade G, D614G (S) [46]. The inoculum was removed 1.5 h later, and DMEM with 2% FBS (with or without BF 1.2) was maintained at 37 °C and 5% CO_2_ for 48 h. All experiments using SARS-CoV-2 were performed under biosafety level 3, following containment procedures approved at the Laboratory of Virology Unit, Pisa University Hospital.

#### 2.5.4. High-Content Confocal Imaging

Imaging experiments were performed using an Operetta CLS high-content imaging device (PerkinElmer, Hamburg, Germany) and were analyzed with Harmony software version 4.6 (PerkinElmer, Hamburg, Germany). Huh-7 and Vero-TMPRSS cells were seeded in 96-well CellCarrierUltra plates (Perkin Elmer, Hamburg, Germany) 24 h prior to treatment or infection. Cells were fixed in paraformaldehyde, 4% in PBS and stained 48 h after infection with Anti-SARS-CoV-2 Spike protein antibody, 1:1000 (#40588 RC02, Sino Biological, Beijing, China). Nuclei were stained with DAPI (1 µg/mL).

### 2.6. Statistical Analysis

Data were analyzed using GraphPad Prism (version 8.01; GraphPad Software Incorporated, San Diego, CA, USA). Statistical significance was established at *p* < 0.05. Kruskal–Wallis tests or Mann–Whitney tests were employed for analyzing data, and individual differences were analyzed using post hoc Dunn’s multiple comparison tests. One-way ANOVA was used for evaluating the biological results.

## 3. Results

### 3.1. Characterization of the Nasal Emulgels

**Figure 2:**
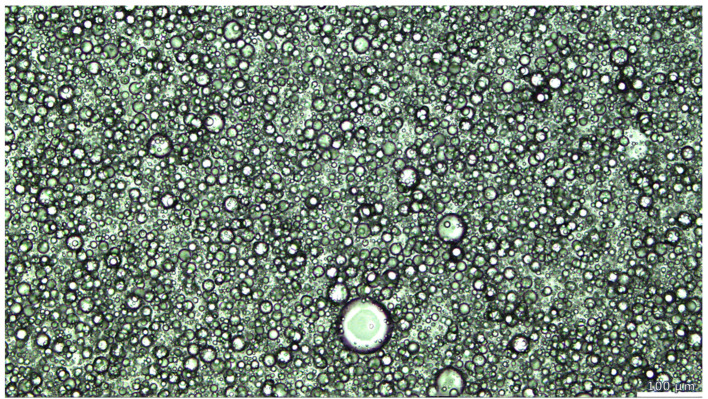
Leica DM6 polarized light microscopy images of BF 1.2.

Morphological analysis (Figure 2) showed that the oil phase performed well and was homogenously distributed in the aqueous network similar to the structure oil in HPMC-based gels, used as drug delivery systems, reported by Corredo-Chaparro et al. [38].

No significant differences (*p* > 0.05) were found in pH values (Table 1) for BF 1, BF 1.2 or BF 1.5. Moreover, Shmuel et al. [12] reported the importance of low pH HPMC-based nasal spray such as Taffix to reduce SARS-CoV-2 infections by creating a local acid microenvironment (e.g., pH 3.5) on human mucosa, like that demonstrated for respiratory viruses like N1H1 influenza and Rhinoviruses [47,48]. Nevertheless, the use of nasal formulations with pH around 3.5–3.7 was considered to be safe and well tolerated as recently described in a Nasus Pharma report [49].

Viscosity (Table 1) was used to estimate the suitability of the three formulations for nasal administration. According to the literature, the viscosity was proportional to the amount of HPMC [36,50]. Taking into account this parameter, BF 1.2 was selected as the most suitable since values around 1000 cp have been considered acceptable for intranasal applications [51].

Spreadability results demonstrated the inverse correlation with the percentage of HPMC since the emulgel with the lowest value was BF 1.5. Statistical analysis, evaluating how the increase in percentage of HPMC affected viscosity and spreadability, highlighted a significative difference among the three formulations, giving values of *p* < 0.05 and *p* < 0.05, respectively. This result confirmed the straight correlation between viscosity and spreadability, as previously explored by mathematical models [52]; viscosity was employed as a predictor parameter of the spreadability behavior, and was found to be the most impacting factor.

**Figure 3:**
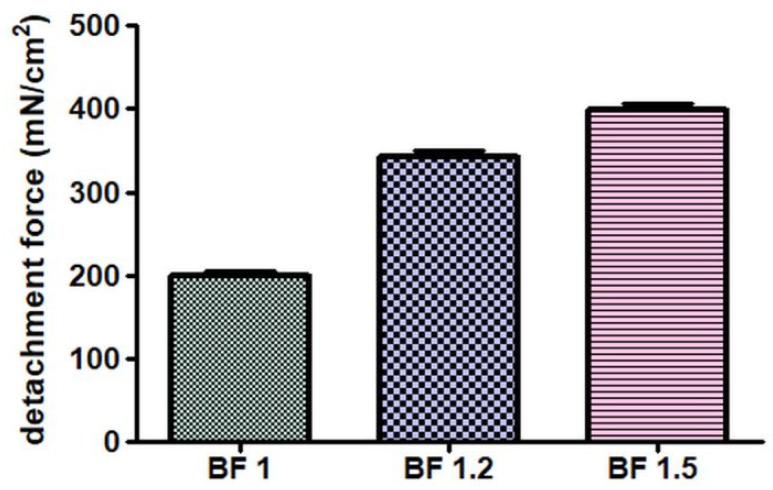
Mucoadhesion strengths for BF 1, BF 1.2 and BF 1.5.

Experimental results of mucoadhesive strength are graphically reported in Figure 3. The strongest value was obtained by the analysis of BF 1.5, due its ability to form entanglements and Van der Waals forces because of the polymer concentration [40]. These results satisfied the need for adequate mucoadhesion that can be used as a fundamental physicochemical parameter for nasal formulations. However, good in vitro mucoadhesive values were also obtained with BF 1.2, in line with results obtained for polymeric formulations that are useful for prolonging the residential time of the formulation in the nasal cavity [42]. The Kruskal–Wallis test on the experimental data resulted in *p* < 0.0001, demonstrating the significance of the percentage of HPMC in mucoadhesion properties. The amount of bioadhesive polymer has been demonstrated to play a key role in the preparation stage: during the mucoadhesion process, the formulation spreads over the surface and increases the substrate contact by the interaction of its chains within the mucus [53]. In general, the mechanism of mucoadhesion has been described as a two-step process based on the contact stage and the consolidation stage. During the consolidation steps, Van der Waals forces are very important since even if considered weak, a great number of interactions leads to intense global adhesion [53]. In addition, studies on the nasal spray Taffix demonstrated that its barrier action against the SARS-CoV-2 viruses was based on the reinforcement of the bioadhesive forces related to the use of the HPMC gel, which limit the engage with the receptors and the penetration in human cells [12,48].

### 3.2. Physical Appearance and Stability

The evaluations of physical stability of BF 1, BF 1.2 and BF 1.5 demonstrated no significant differences (*p* > 0.05) in any of the checked parameters, independently of the percentage of HPMC in the composition, providing the stability of the emulgels during the tested period (Table S1). Nevertheless, no changes in the color of the three emulgels over 28 days were noticed (Figure S2). On the other hand, it was noticed that the three emulgels showed phase separation after 1 day, although they returned to their initial appearance after simple manual agitation.

### 3.3. Biological Assays

**Figure 4:**
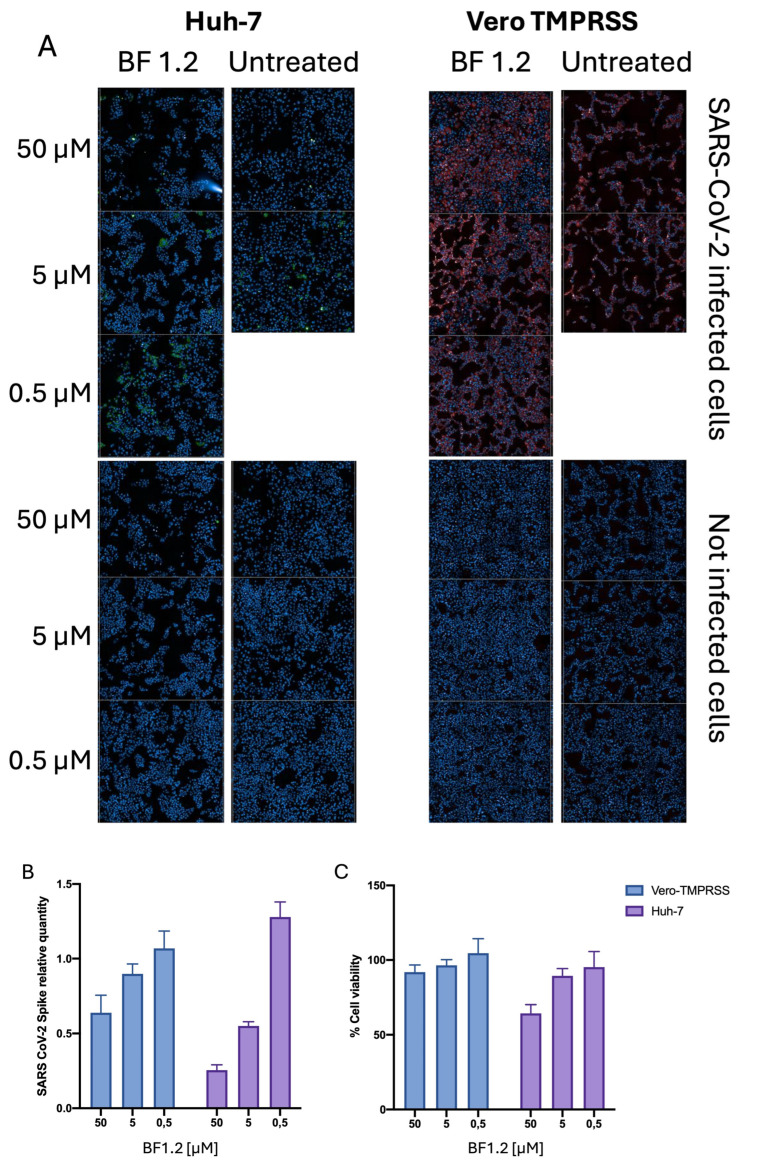
Quantification of SARS-CoV-2 infection and cell viability upon BF 1.2 treatment. (**A**) High-content confocal microscopy analysis of SARS-CoV-2-infected cells. SARS-CoV-2 Spike protein was quantified in infected cells at 48 h after infection, treated or untreated with BF 1.2. Blue: DAPI; left: Huh.7, green: spike; right: Vero-TMPRSS, red: spike. (**B**) Quantification of spike protein in relation to infected, untreated cells. (**C**) Cell viability upon treatment with various concentrations of BF 1.2, calculated based on nuclei count of cells present in treated and untreated wells. Data are expressed as means ± SD (*n* = 3).

Biological evaluations of BF 1.2, selected as the leading emulgel, were carried out on two different cell lines, with the aim of evaluating its antiviral properties. SARS-CoV-2 infection occurs by binding to the ACE-2 receptor with the help of TMPRSS; both are naturally present on Huh-7, whereas Vero cells only have ACE-2 and were stably transfected with TMPRSS. After incubation for 48 h after infection with SARS-CoV-2 with or without BF 1.2, quantification of Spike viral protein demonstrated a reduction in viral expression (Figure 4).

Cytotoxicity was measured for BF 1.2 in Huh-7 cells and Vero-TMPRSS and CC50 resulted in 334.00 ± 1.81 µM and 38.00 ± 1.98 µM, respectively.

As can be seen in Figure 4A, untreated infected cells exhibit marked cytopathic effects and cell lysis; therefore, although there is less spike fluorescence due to the lower number of cells, more cells show expression of spike protein, as a sign of a greater infection degree. As shown in Figure 4, in vitro, BF 1.2 significantly disrupted the cell-to-cell transmission pathway utilized by SARS-CoV-2—an efficient and stealthy mechanism that facilitates rapid viral propagation [54]. This inhibition may have important implications for SARS-CoV-2 transmission, particularly in the early stages of infection. By limiting viral expansion after initial infection, BF 1.2 could potentially reduce disease onset and curb transmission to others. Currently, the mechanism by which the gel acts as a barrier to cell-to-cell spread is unknown; it may simply function as a physical barrier due to the properties of the emulgel or its components. Future experiments will focus on evaluating its potential to block viral entry and the effect of the emulgel on ciliated cells. Indeed, it may also act as a barrier to infection itself but this condition could not be tested in our cell model. BF 1.2 may function similarly to Iota-Carrageenan nasal spray, which has shown in vivo protective and preventive effects against COVID-19 when used as prophylaxis both after and during exposure to SARS-CoV-2 [16]. The proposed mechanism involves interaction with the viral particle surface, thereby inhibiting viral entry into host cells and capturing particles released from infected cells [16,54,55]. Therefore, BF 1.2 could be proposed as a simple, safe and effective barrier emulgel for the prevention and control of acute respiratory tract infections. Future experiments will focus on evaluating the mechanism and, in particular, its potential to block viral entry as well the effect of the emulgel on infected ciliated cells. After this, the emulgel’s ability to deliver an antiviral drug with local effects could be evaluated in order to enhance the antiviral activity.

## 4. Conclusions

This study demonstrates that an effective nasal mucoadhesive emulgel can be developed using readily available, safe, and low-cost excipients. By properly modulating the concentration of HPMC, the emulgel BF 1.2 was identified as optimal, exhibiting suitable mucoadhesive strength, viscosity, and spreadability for intranasal administration. In vitro assays confirmed that BF 1.2 acts as a simple and effective barrier to SARS-CoV-2 diffusion, supporting its potential as a prophylactic strategy against respiratory viral infections. The use of biocompatible and economical excipients enhances the formulation’s feasibility for rapid clinical translation and large-scale production. Future experiments will focus on the evaluation of a barrier to viral entry, and potential enhancement of antiviral efficacy through the incorporation of lipophilic antiviral agents. Overall, this novel emulgel represents a practical and scalable pharmaceutical platform with promising applications in infection prevention and pandemic preparedness.

## Figures and Tables

**Table 1:** pH, viscosity and spreadability of emulgels (mean ± SD; *n* = 9).

	HPMC (%)	pH	Viscosity (cp)	Spreadability (cm)
BF 1	1	3.9 ± 0.1	212 ± 11	8.37 ± 0.12
BF 1.2	1.2	3.8 ± 0.2	1080 ± 83	7.27 ± 0.06
BF 1.5	1.5	3.7 ± 0.1	2884 ± 11	6.43 ± 0.12

## Data Availability

The raw data supporting the conclusions of this article will be made available by the authors on request.

## Supplementary Materials

The following supporting information can be downloaded at: https://www.mdpi.com/article/10.3390/pharmaceutics17060795/s1, Figure S1: Illustrative scheme for the spreadability method. The dotted line shows the increase in diameter used for the measurement.
