# Supplementary material for: Nasal Emulgel’s Role in Preventing Coronavirus Infection
Source: Pharmaceutics. 2025 Jun 19;17(6):795. doi: 10.3390/pharmaceutics17060795 (PMC12197114; doi:10.3390/pharmaceutics17060795)
Supplement: Supplementary file 1 [file pharmaceutics-17-00795-s001.zip › pharmaceutics-3680212-supplementary.pdf]

pharmaceutics

SUPPLEMENTARY MATERIAL

Nasal Emulgel's Role in Blocking Coronavirus Infection

Francesca Accioni [1], Giovanna Rassu [1,*], Antonio Brunetti [2], Erika Plicanti [3,4], Giulia Freer [4], Antonio Carta [1], Paolo Giunchedi [1] and Elisabetta Gavini [1]

[1] Department of Medicine, Surgery and Pharmacy, University of Sassari, 07100 Sassari, Italy; francesca.accioni@gmail.com; acarta@uniss.it; pgiunc@uniss.it; eligav@uniss.it
[2] Biomedical Sciences Department, University of Sassari, 07100 Sassari, Italy; brunetti@uniss.it
[3] Department of Medical Biotechnologies, University of Siena, 53100 Siena, Italy; e.plicanti@studenti.unipi.it
[4] Centro Retrovirus, Department of Translational Research, University of Pisa, 56127 Pisa, Italy; giulia.freer@unipi.it
* Correspondence: grassu@uniss.it.

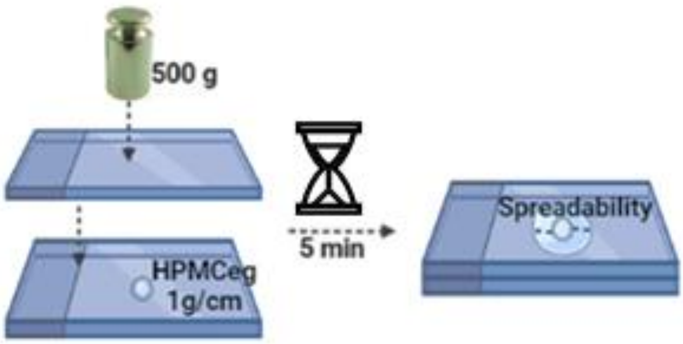

Figure S1 Illustrative scheme for the spreadability method. The dotted line shows the increase in diameter used for the measurement.

pharmaceutics

MDPI

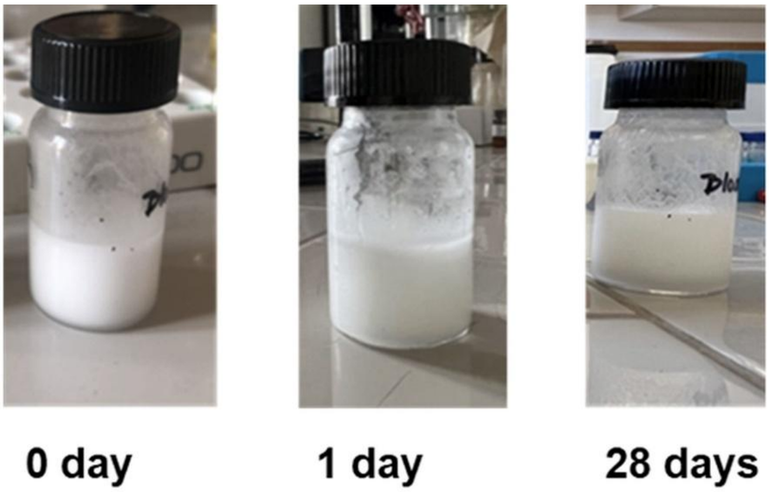

Figure S2 Physical appearance of BF 1.2, representative of the three emulgels, within 28 days of storage at 25 °C. For imaging, BF 1.2 was transferred into a clear glass vial to allow visual assessment.

pharmaceutics

Table S1 pH and viscosity of the three emulgels within 28 days of storage at 25 °C.

		Day 7		Day 14		Day 21		Day 28	
		mean	SD	mean	SD	mean	SD	mean	SD
BF 1	pH	3.7	0.1	3.9	0.1	3.9	0.1	3.9	0.1
	Viscosity (cP)	191.5	3.1	229.0	15.6	215.3	4.2	215.7	11.0
BF 1.2	pH	3.8	0.0	3.9	0.1	3.9	0.1	3.9	0.1
	Viscosity (cP)	1161.7	55.5	1162.7	67.9	1164.7	68.7	1146.3	55.9
BF 1.5	pH	3.8	0.0	3.8	0.0	3.9	0.1	3.9	0.1
	Viscosity (cP)	2488.7	57.7	2544.7	142.1	2431.0	131.0	2400.7	77.8
